# Stable organization of the early lexical-semantic network in 18- and 24-month-old preterm and full-term infants: an eye-tracker study

**DOI:** 10.3389/fpsyg.2023.1194770

**Published:** 2023-09-21

**Authors:** Anett Ragó, Zsuzsanna Varga, Miklos Szabo

**Affiliations:** ^1^Department of Psychology, Faculty of Health Sciences, UiT The Arctic University of Norway, Tromsø, Norway; ^2^Department of Cognitive Psychology, Eötvös Loránd University, Budapest, Hungary; ^3^Division of Neonatology, Department of Pediatrics, Semmelweis University, Budapest, Hungary

**Keywords:** lexical-semantic network, eye-tracking method, taxonomic versus associative relations, late preterm toddler, priming, visual world paradigm

## Abstract

**Introduction:**

An organized mental lexicon determines new information acquisition by orienting attention during language processing. Adult-like lexical-semantic knowledge organization has already been demonstrated in 24-month-olds. However, the outcomes of earlier studies have been contradictory in terms of the organizational capacities of 18-month-olds, thus our aim was to examine lexical-semantic organization in this younger age group. In prematurely born infants, audiovisual integration deficits have been found alongside disruptions in language perception. By including late preterm infants with corrected ages in our study, we aimed to test whether maturational differences influence lexical-semantic organization when vocabulary is growing rapidly.

**Methods:**

We tested 47 late preterm and full-term 18- and 24-month-old infants by means of an infant-adapted target-absent task using a slightly modified version of the original visual world paradigm for eye tracker.

**Results:**

We found a longer fixation duration for the lexical and semantic distractors compared to the neutral pictures. Neither language proficiency nor age affected the looking time results. We found a dissociation by age between taxonomic and associative semantic relations. Maturational differences were detectable in the initial processing of taxonomic relations, as processing in the preterm group was slightly delayed and qualitatively different in the first half of the looking time. The size and composition of the expressive vocabulary differed only by age.

**Discussion:**

In general, our study demonstrated a stable lexical-semantic organization between 18 and 24 months of age, regardless of maturational differences.

## 1. Introduction

In parallel with the development of their segmentation ability, infants learn to connect lexical information (sound contrast) to objects in the outside world. The establishment of the early lexical-semantic network starts with the recognition of the sound pattern of some frequent words at around 4.5 months of age (Johnson, 2016). Initially, the early proto-lexicon includes references for only the most frequent word forms (such as *mommy* or *daddy*) and is highly specialized to specific tokens (infants do not generalize these words to similar items at this point). As the infant’s vocabulary increases after the first year of life, the entries need to be systematically organized. Earlier studies, using an infant-adapted auditory-visual priming paradigm [the so-called intermodal preferential looking (IPL) paradigm], have demonstrated the semantic organization of the knowledge structure from 21 months of age (Arias-Trejo and Plunkett, 2009). Similarly, an electrophysiological study found an N400-like priming effect for a semantic word category change in 24-month-old Norwegian toddlers (von Koss Torkildsen et al., 2007), while another study found similar results in French 24-month-olds (Rämä et al., 2013). By contrast, we have only contradictory results when it comes to knowledge network organization before 20 months of age. The question thus arises as to whether 18-month-old infants, at the beginning of the vocabulary spurt, acquire their first words in semantic isolation, or whether a methodological inconsistency has given rise to the contradictory results found in the case of younger age groups (cf. Wojcik, 2018).

In the present study, we aimed to identify differences in knowledge system organization between 18 and 24 months of age. Our aim was to investigate whether a similar sensitivity to lexical and semantic relations as that found in earlier studies in 24-month-olds (von Koss Torkildsen et al., 2007; Rämä et al., 2013; Chow et al., 2017) exists in the younger age group. To test the relevance of language proficiency (the size and composition of the expressive vocabulary) in the development of organization, we included an at-risk group in the study. We recruited healthy, late preterm infants (born between 34 and 36 weeks of gestational age), as their language perception capacity develops later and differently compared to that of their peers (Varga et al., 2021). We hypothesized that the qualitative differences in early vocabulary between preterm and full-term infants might be responsible for the possible organizational differences. Comparisons based on age and gestational status enabled us to understand the maturation of early semantic system organization from 18 to 24 months and the possible factors behind the differences in organization.

The infant-adapted IPL paradigm applies the principle of the lexical decision paradigm, where semantic information shortens the lexical decision time for the subsequent item (Meyer and Schvaneveldt, 1971). In the infant-adapted version, the idea is that by looking longer at the semantic or phonological distractor compared to the non-related items, infants demonstrate the lexical-semantic organization of the early knowledge system. Styles and Plunkett (2009) developed the first version of the infant-adapted IPL paradigm using a prime word followed by the target word. Participants then saw two pictures showing the target and a phonological distractor. The (semantic) relation between the prime and target words varied. In Styles and Plunkett (2009) study, the 18-month-olds produced preferential looking for the named target picture, independently of the prime. In the case of the 24-month-olds, however, the target preference was modulated by the semantic relatedness of the prime. In their discussion of the results, the authors emphasized the greater variability in the looking time data in the case of the younger age group.

A similar study (Arias-Trejo and Plunkett, 2009) tested 18- and 21-month-old infants using a modified IPL paradigm. Besides the prime–target condition, the authors introduced three other conditions to test the participants’ sensitivity to semantic relatedness in the presence and absence of the target word. The 18-month-olds showed visual target preference in the named target conditions (prime–target: *cat*–*dog*; and neutral–target: *bike*–*dog*). The 21-month-olds, however, differentiated between the two conditions and did not look longer at the target picture in the case of an unrelated prime word (*bike*–*dog*). The authors interpreted this as the presence of an interference effect in 21-month-olds, as a result of which the processing of the prime word distracted them from processing the target word. This phenomenon is well documented in adults (Neely, 1976). However, even the 21-month-olds failed to detect the semantic relation between the prime word and the target picture in the prime–“look” condition. Furthermore, in this paradigm, the distractor phonologically matched the target word and not the prime (*dog*–*door*; here, the prime was the word *cat*). We know that spoken word recognition includes the mapping of speech sounds and the automatic activation of competing phonological representations (Allopenna et al., 1998). Thus, there was a contrast between the prime and the two picture labels, which probably confused the infants. This latter study (presenting three, slightly modified experiments) demonstrated how different factors (stimulus onset asynchrony, the familiarity of the words, the nature of the semantic relation, and the characteristics of the distractor word/picture) might easily influence the results.

At the same time, convincing results have been obtained with respect to the abilities of 18-month-olds to recognize and implicitly name a picture of a familiar object (after looking at a picture of a cat, they looked longer at a cap than at a shoe) (Mani and Plunkett, 2010). Furthermore, lexical organization is well demonstrated in this age group, as the phonological distractor (*cup*) attracted longer looking times even in the absence of the target word (*cat*). A later study revealed the capacity of 18-month-olds to detect the semantic relatedness of acoustically presented words (Delle Luche et al., 2014). This head-turn preference study clearly demonstrated that at 18 months of age, infants already process the taxonomic relations of words (i.e., they process the meaning of the words and have semantic organization). A recent study revealed a semantic facilitation effect in 18-month-olds (Borovsky and Peters, 2019). Based on the early receptive vocabulary of infants and adult association norms, Hills et al. (2009b) modeled the graph-theoretic properties of the development of the early lexical-semantic network from 16 to 30 months of age. They contrasted different predictive network growth hypotheses based on adult data and concluded that early vocabulary growth is based on lexical and semantic associations (see also Hills et al., 2009a). We argue that there is no direct evidence for different lexical-semantic organization before 20 months of age, thus the contradiction might be resolved by the new paradigm and a systematic comparison of different developmental groups.

To directly measure early knowledge representation differences, it is important to consider not only the vocabulary of the participants (their familiarity with the words) but also the type of semantic relation. Studies that have identified differences among 18-month-olds based on the size of their expressive vocabulary (Arias-Trejo and Plunkett, 2009; Rämä et al., 2013) used both associative and taxonomic relations. In 24-month-olds and older toddlers, researchers have found sensitivity to thematic (associative) relations (Chow et al., 2017). Both taxonomies (classifications according to kind) and partonomies (classifications according to parts) are organizing principles for categories (Tversky, 1989). At the basic level, taxonomies can be constructed by means of the similarity of the members, making them easy for infants to acquire (Murphy, 2018). The acquisition of early perceptual categories by exposure to the members of two contrasting categories starts at 3 months of age (Quinn et al., 1993, 2001; Quinn and Eimas, 1998). Although these categories are still fragile and their boundaries depend on the stimulus set (Behl-Chadha, 1996), they form the basis of the adult categories. Partonomies are based on the detection of similarly functioning parts of objects or situations, thus they are referred to as thematic or associative relations. Waxman and Hall (1993) demonstrated that a preference for taxonomic relations over thematic relations exists from 15 months of age. They also suggested that thematic preference appears later in the course of development (but not before the 21st month). Tversky (1989) also demonstrated that sensitivity to functional parts appears later during development, facilitated by the recognition of functional similarity at the superordinate level. Hills et al. (2009b) found that, unlike adults, infants build their mental lexicon according to the specificity of the learning environment (that is, the relatedness of the words they hear in everyday conversations). A recent analysis of adult L2 learners’ semantic networks also confirmed the dominance of taxonomic relations in the early stages of acquisition (Agustín-Llach, 2023). We argue that experience helps in building more complex thematic categorical associations. As a consequence, we predicted a more stable knowledge of taxonomic categories in 18-month-olds in the case of animates, and a more developed associative knowledge in 24-month-olds.

Chow et al. (2017) modified the IPL paradigm, adapting an existing adult visual world paradigm to infants (Huettig et al., 2011). Here, the priming situation was retained and the focus was rather on audiovisual integration capacity. In the target-absent task, the target word was presented acoustically and was followed by the visual presentation of four distractors: one semantic, one phonological, and two unrelated. The authors tested 24- and 30-month-old infants to assess their sensitivity to lexical and semantic relations. The eye-tracking device made it possible to capture the dynamics of the infants’ eye movements and to follow the direction of their gaze throughout the presentation of the visual items. The results confirmed adult-like lexical-semantic organization in both age groups. The authors emphasized the role of acoustic input in orienting visual searches for meaningful objects during knowledge acquisition.

The integration of acoustic and visual modalities during speech perception (temporal alignment) plays an important role in speech processing in both adults and infants (Navarra et al., 2012). Early audiovisual speech integration may be particularly complicated for preterm infants, as they spend many weeks in incubators in neonatal intensive care units, where the percentage of linguistic stimuli is quite low, while medical device noise and lights are more prominent (Caskey et al., 2011). According to the study by Pickens et al. (1994), preterm infants (with an average gestational age of 34 weeks of gestation) did not show a visual preference for face–voice synchrony at 3, 5, and 7 months of age in the preferential looking task. The study suggested that preterm birth may affect the audiovisual integration process at a very young age. Imafuku et al. (2019) found similar results with preterm toddlers born between 23 and 35 weeks of gestation: the participants did not detect the congruence between speech sounds and lip movements at 6, 12, and 18 months of age. Berdasco-Muñoz et al. (2019) demonstrated that audiovisual speech perception is affected even in late preterm infants. Comparing a native versus a non-native language, they found that the visual scanning of a talking face differed in full-term infants; however, preterm infants showed similar scanning patterns for both languages. We still do not know whether the linguistic aspects of audiovisual processing are also affected in preterm groups, nor do we know whether early differences remain in later stages of development.

In the present study, we used the four-picture, target-absent visual world paradigm in 18- and 24-month-old toddlers to test the activation of phonological and semantic representations during the word recognition process. In our paradigm, we used frequent nouns familiar to both age groups. After the spoken target word, we showed a four-picture array comprising a phonological distractor (realized by the matching onset syllable), a (taxonomic or associative) semantic distractor, and two neutral distractors. To reduce confusion when detecting semantic relatedness, we applied taxonomic relations in those categories (e.g., animate kinds: living things and vehicles), which, at the basic level, thanks to the perceptual features shared among their diverse members, provoke superordinate categorization, while we tested thematic relations using manipulable objects (clothes and furniture). We measured the total duration of fixations on the four possible areas of interest. To better understand the cause of the possible developmental differences, we also tested two age-matched groups of late to moderate preterm infants with corrected ages. The (quantitative and qualitative) vocabulary specificities of the participants were compared using the Hungarian version of the MacArthur-Bates Communicative Development Inventory (CDI; Kas et al., 2017), which contains the most widely used parent-report measures of early language development for both typically and atypically developing children (Hills et al., 2009a; Rämä et al., 2013; Delle Luche et al., 2014; Imafuku et al., 2019).

First, we hypothesized that the lexical-semantic organization of the knowledge system would be found in the full-term 18-month-old group (H1). We predicted that the syllable match between the target word and the phonological distractor in a fixed-stress language like Hungarian would facilitate lexical processing, while the basic-level animate taxonomies and the functional associative relations of familiar objects would facilitate semantic processing (cf. Willits et al., 2013). Accordingly, we predicted longer looking times for the phonological and categorical distractors compared to the unrelated pictures at both 18 and 24 months of age. In addition to the classic, static looking time (fixation duration) calculations, we analyzed the time course patterns in the two age groups and clinical/control groups to reveal possible processing differences in the first half of the exposure time. Based on the study by Chow et al. (2017), we predicted a dissociation by time in reactions to the phonological versus semantic associations, the former being built up faster than the latter. Our second hypothesis (H2) predicted a difference in sensitivity to the semantic relations in the full-term groups. Earlier studies found an associative (thematic) categorical preference at 24 months (Chow et al., 2017). Accordingly, we hypothesized (H2a) that a longer looking time would be registered for the thematic categorical distractors at 24 months. Since infants need experience to represent thematic relations, while basic-level taxonomic relations are present from the early months, we predicted a greater sensitivity to taxonomic relations in the 18-month-old group (H2b). Despite earlier extensive examination of full-term infants’ audiovisual speech integration, the present study is the first examination of audiovisual speech integration in preterm infants using a language priming paradigm. One intriguing question is how this different early environmental stimulation influences preterm infants’ audiovisual speech integration and, consequently, the early organization of the mental lexicon. If the characteristics of language representation (the size and composition of common nouns, predicates, and grammatical function words) influence early knowledge organization, it should be present in healthy late preterm infants, especially at 18 months. Our third hypothesis (H3) was that preterm toddlers’ sensitivity to phonological and semantic relations would not be as strong as that of full-term toddlers at the same maturational age (the preterm toddlers’ ages were corrected to the expected date of delivery). Therefore, we predicted smaller, or no, effects for both semantic and categorical distractors in both preterm groups compared to their full-term peers. We planned to test the effect of vocabulary size (measured using the CDI) on the looking time results. We predicted that—in line with earlier findings (Arias-Trejo and Plunkett, 2009; Styles and Plunkett, 2009)—vocabulary size would not influence lexical-semantic network organization in the older group. We also expected to find qualitative differences in early vocabulary between preterm and full-term infants. By introducing the latest infant-adapted priming experimental technique compatible with eye tracking, our results can identify whether an adult-like lexical-semantic network typically exists from the onset of the language spurt.

## 2. Materials and methods

### 2.1. Participants

Forty-seven toddlers participated in our study: 24 full-term toddlers and 23 preterm toddlers. Table 1 summarizes the characteristics of the participants. There were 13 toddlers in the group of 18-month-old full-term infants (FT18) and 11 toddlers in the group of 24-month-old full-term infants (FT24). The preterm (PT) toddlers were measured at 18 months of corrected age (PT18; *n* = 10) and at 24 months of corrected age (PT24; *n* = 13). The preterm and full-term groups were matched according to their maturational ages. To validate our group selection, we compared the age (in the case of the PT infants, we used the corrected age), birth weight, and gestational age of the FT18 and PT18 infants [Age: *F*(1,21) = 0.564, *p* = 0.46; Birth weight*: F*(1,21) = 22.08, *p* < 0.001; Gestational age: *F*(1,21) = 87.52, *p* < 0.001], as well as the age, birth weight, and gestational age of the FT24 and PT24 infants [Age: *F*(1,22) = 0.58, *p* = 0.45; Birth weight: *F*(1,22) = 10.31, *p* < 0.001; Gestational age: *F*(1,22) = 38.4, *p* < 0.001]. The PT and FT groups were also matched according to their socioeconomic status.

**TABLE 1:** Characteristics of the participants.

	Mean chronological age in months (SD)	Mean corrected age in months (SD)	Mean birth weight in grams (SD)	Mean gestational age in weeks (SD)
FT18 (*N* = 13)	18.25 (0.5)	–	3,517 (533.8)	38.9 (1.25)
PT18 (*N* = 10)	19.62 (0.71)	18.34 (0.78)	2,457 (520.3)	35.1 (0.329)
FT24 (*N* = 11)	24.48 (1.08)	–	3,165 (463.236)	38.3 (1.75)
PT24 (*N* = 13)	25.46 (0.4)	24.27 (0.36)	2,481 (562.91)	34.8 (0.98)

Inclusion criteria were (1) normal hearing; (2) normal scalp ultrasound; (3) monolingual Hungarian family; (4) appropriate birth weight for gestational age; (5) no chromosomal malformations; and (6) no asphyxia in the anamnesis (Supplementary Table 1 summarizes the general clinical characteristics of the participants). Ten additional toddlers were tested and excluded from the final analysis due to fussiness (*n* = 2), technical problems (*n* = 1), attention deficit (*n* = 3), or inappropriate birth weight for gestational age (*n* = 4).

The participating families were recruited from the Neonatal Intensive Care Unit, 1st Department of Pediatrics, Semmelweis University, and the Neonatal Intensive Care Unit, 2nd Department of Obstetrics and Gynecological Clinic, Semmelweis University. The parents of the participating toddlers gave their written consent to the experiment in accordance with the World Medical Association Declaration of Helsinki before the data were collected. The study protocol was approved by the local ethical review board of Eötvös Loránd University (ref. no: 2019/267).

### 2.2. Material

**FIGURE 1:**
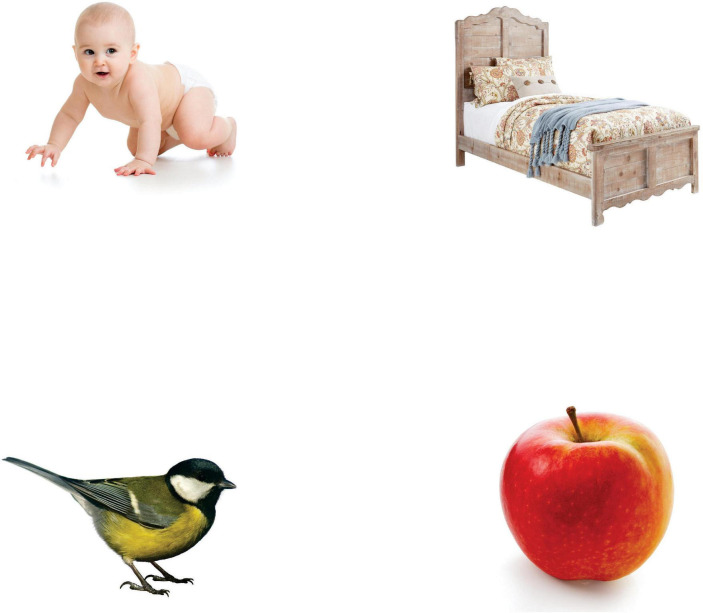
Example of a four-picture array (Panel 1). In Panel 1, the target word is *banán* (banana); the phonological distractor is *baba* (baby) at position 1; the categorical distractor is *alma* (apple) at position 4; and the unrelated distractors are *ágy* (bed) and *madár* (bird), at positions 2 and 3. Image source: (top-left) Oksana Kuzmina/Shutterstock.com, (top-right) JeweBewe/Shutterstock.com, (bottom-left) Maksym Gorpenyuk/Shutterstock.com (bottom-right) Karlisz/Shutterstock.com.

The test material comprised 11 panels, with four pictures on each, arranged in a square matrix (see Figure 1 showing a realistic version of panel 1). The standard panel size was 1,362 × 1,185 pixels (1.61 Mpixels), which equals 23.1 × 20.1 cm. The four square-shaped pictures were positioned in the upper left (position 1), upper right (position 2), lower left (position 3), and lower right (position 4) corners of an invisible square, with 6 cm between each picture horizontally and vertically. Each panel was paired with an acoustic stimulus: the label of an everyday object (target word: TW). The panels showed three types of pictures: a phonological distractor (PD), a categorical distractor (CD), and two unrelated distractors (to achieve a symmetrical arrangement, we used two of these pictures: U1 and U2). The phonological distractor was an item with a label that started with the same onset syllable as the TW. The CD pictures were separated into two types, depicting either taxonomic or thematic relations. We used taxonomic relations in the case of animate kinds (so-called living things, and one set of vehicles), and thematic relations in the case of inanimate kinds (objects and furniture). Supplementary Table 2 presents the names and category types of the objects used. The position of the three types of pictures varied systematically on each panel, producing a definite order of panels with a different starting panel in the case of each participant. The pictures were chosen from the Hungarian edition of Bayley Scales of Infant and Toddler Development–Third Edition (Bayley, 2006) and the “First Words” card set for children (published by Peak Fun). The pictures and target words denoted simple, everyday objects (nouns) familiar to infants at the tested age, as in earlier studies (von Koss Torkildsen et al., 2007; Hills et al., 2009a; Rämä et al., 2013; Mayor and Plunkett, 2014; Chow et al., 2017).

To study the size and composition of the lexicon at 18 and 24 months of age, we used a parental rating method, the Hungarian version of the MacArthur-Bates CDI (Kas et al., 2017). The Hungarian version contains 798 items presented in 22 categories (words and sentences). A detailed description of the CDI categories is provided in Supplementary Table 3. The composition analyses focused on four categories: social terms, common nouns, predicates, and grammatical function words. The categorization system used in the present study was the same as that used in other studies on the composition of the early lexicon in other languages (Jackson-Maldonado et al., 1993; Maital et al., 2000; Koster et al., 2005).

### 2.3. Procedure

In each panel presentation, the participants were exposed to a video starting with an attention-grabbing sound with an accompanying visual effect, followed by a female native speaker calling the attention of the child (“*Figyelj csak!*”/“Listen!”) before uttering the TW. The 3-s video was followed by the specific panel related to the TW. Each panel was presented for 2,500 ms post label onset. During the whole session, we registered the eye movements of each participant as they sat in their parent’s lap, approximately 65 cm from the computer screen. The participants’ eye movements were captured by the Tobii T60 XL Eye Tracker, and the stimuli were presented using Tobii Pro Studio, which synchronizes stimulus presentation and eye gaze recording.

### 2.4. Measurements and statistical analyses

In the case of the CDI categories, the vocabulary production total scores were calculated by adding up the category scores. For fixation identification, we used the velocity-threshold fixation identification (I-VT) classification algorithm of the Tobii Pro Studio software, which classifies eye movements based on the velocity of the directional shifts of the eye (Olsen, 2012). We created areas of interest (AoI) around the pictures on the panels. In the case of the unrelated pictures, we calculated the average of the two fixation indices. We analyzed the mean total fixation duration (the mean of the total fixation durations on the AoIs) and the fixation probability for specific AoIs along time. The former was measured for the total exposure to the panels (2,500 ms), while the latter statistical analyses were completed for the first half of exposure post label onset (0–1,000 ms), following the method used by Chow et al. (2017). To test the role of language proficiency in the fixation durations, we applied linear regression for the PD, CD, and U picture types.

The 60 Hz sampling rate of the eye-tracking device registers fixation with a sampling interval of approximately 8.3 ms for valid fixations. Invalid fixations (i.e., fixations outside the screen, blink-related errors, or gaze) were excluded from the analysis. We obtained a valid fixation for each panel at every 200 ms on average. Based on this, we calculated the proportion of fixations in each 200 ms time bin for the three picture types (PD, SD, and U). To detect the appearance of organization over time, we analyzed the first half of the total exposure time from visual panel onset (0–1,000 ms). To compare the looking patterns of the age groups and clinical status groups over a few time bins in the first half of the exposure, we adopted the conventional approach of a mixed ANOVA (cf. Huang and Snedeker, 2020; McMurray, 2022). To avoid false interaction effects caused by the non-linearity of the probability data, we applied logistic transformations to the proportions, as suggested by Barr (2008). In the mixed-design ANOVA, when the sphericity assumption was violated, we used the Greenhouse-Geisser correction. When repeated comparisons were needed, we used the Bonferroni correction. For the statistical analyses, we used IBM SPSS Statistics 26 Software.

### 2.5. Results

To identify the language performance differences between the groups based on the CDI, we ran a multivariate analysis of variance for the CDI vocabulary total score and the four subscale scores in each of the two groups (preterm vs. full-term and 18 vs. 24 months of age). According to the results, the group of 24-month-olds outperformed the group of 18-month-olds in all scales, independent of gestational status. We did not find any main effect for gestational status, thus the preterm infants did not perform worse than their full-term peers. Age and gestational status interaction was significant in the case of social terms. This interaction was caused by the fact that the preterm versus full-term difference was present at 18 months but was absent at 24 months (For a detailed description, see Supplementary Table 4). Table 2 presents the median for CDI total score and subscale scores in the age and clinical status groups.

**TABLE 2:** Vocabulary production total score and the median of the CDI subscale scores (min–max) in the age versus clinical status groups.

Total score and subscale scores of the CDI		PT toddlers (min–max)	FT toddlers (min–max)
Vocabulary total score	All	37 (0–524)	57 (13–570)
	18 months	11 (0–42)	35 (13–431)
	24 months	316 (35–524)	274 (17–570)
Social terms	All	23 (0–62)	26 (8–71)
	18 months	5 (0–17)	20 (8–44)
	24 months	43 (22–62)	41 (14–71)
Nouns	All	15 (0–300)	18 (0–298)
	18 months	2 (0–20)	13 (0–267)
	24 months	182 (11–300)	147 (3–298)
Predicates	All	0 (0–172)	5 (0–193)
	18 months	0 (0–1)	1 (0–86)
	24 months	40 (0–172)	57 (0–193)
Grammatical words	All	4 (0–91)	12 (0–103)
	18 months	0 (0–7)	6 (0–45)
	24 months	26 (0–91)	24 (0–103)

As the positions of the CD and PD pictures were not perfectly balanced (the PD pictures were more often in positions 1 and 4 while the CD pictures were more often in positions 2 and 3), we first tested a possible effect of the positioning of the pictures on the panels (i.e., whether any position attracted more or less attention). We therefore ran a 4 × 2 × 2 mixed ANOVA using age (18 vs. 24 months) and gestational status (preterm vs. full-term) as the grouping variables and using a within-subject variable: the position of the pictures on the panel (1–4). The main effect of position was significant [*F*(3,129) = 7.622, *p* < 0.001, μ^2^ = 0.151]. The contrasts revealed that this effect was caused by longer fixations on the first (upper left) position [*F*(1,43) = 22.61, *p* < 0.001, μ^2^ = 0.35]. The fourth (bottom right) position also attracted (marginally significant) longer fixations (*p* = 0.077), while the second and third positions were less fixated on. To counterbalance the dominance of positions 1 and 4 over 2 and 3, we recalculated the original fixation duration results, applying a correction index determined by the magnitude of the given position effect (position 1/1.143; position 2/0.861; position 3/0.939; position 4/1.053).

**FIGURE 2:**
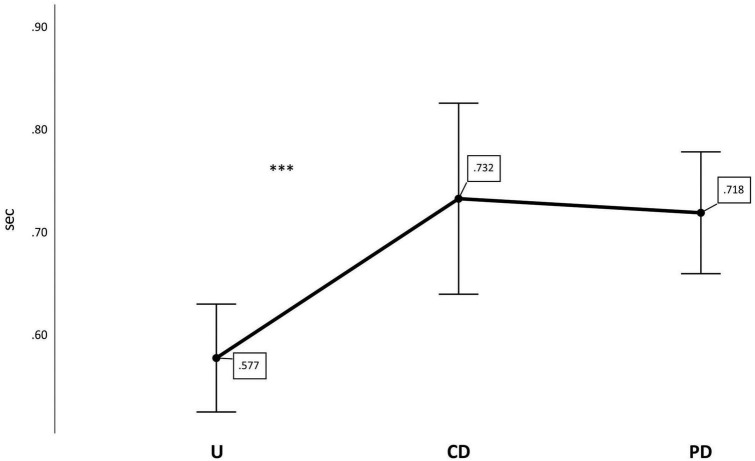
Position-counterbalanced total fixation duration averages per picture type. U (unrelated), CD (categorical distractor), and PD (phonological distractor). Error bars represent ± 2 standard errors of the mean. ***p < 0.001.

To test the effect of picture type (U, CD, PD), we compared the total fixation durations, counterbalanced for position, on the three picture types between the two age groups (18 vs. 24 months) and gestational status groups (preterm vs. full-term) in a 3 × 2 × 2 mixed ANOVA, where the between-subject effects were age group and clinical status and the within-subject variable was picture type (U, CD, PD). For the main effect of picture type, the Greenhouse-Geisser estimate of departure from sphericity was ε = 0.72. This main effect was significant [*F*(1.45,62.13) = 11.943, *p* < 0.001, μ^2^ = 0.217]. The contrasts revealed that the fixation durations on the U pictures were significantly shorter than those on both the CD pictures [*F*(1,43) = 21.193, *p* < 0.001, μ^2^ = 0.33] and the PD pictures [*F*(1,43) = 31.872, *p* < 0.001, μ^2^ = 0.426] (see Figure 2). We found no significant main effect of either age or gestational status. Furthermore, none of the interactions between the variables was significant. In general, the participants looked longer at the PD and CD pictures, independent of age and gestational status.

To test the effect of language proficiency on the position-counterbalanced fixation duration, we ran three linear regressions for the three picture types. The results showed that language proficiency did not predict fixations for any of the picture types (see Table 3).

**TABLE 3:** Linear model of predictors of total fixation durations.

	*b*	SE B	β	*p*
Constant	0.71 (0.64, 0.78)	0.04		0.001
PD	0.004 (−0.02, 0.03)	0.014	0.041	0.784
Constant	0.71 (0.61, 0.83)	0.06	0.001	0.001
CD	0.013 (-0.3, 0.06)	0.02	0.09	0.55
Constant	0.57 (0.51, 0.63)	0.03		0.001
U	0.006 (−0.2, 0.03)	0.12	0.068	0.648

95% bias corrected and accelerated confidence intervals are reported in parentheses. Confidence intervals and standard errors are based on 1,000 bootstrap samples. R^2^ (PD) = 0.002; R^2^ (CD) = 0.008; R^2^ (U) = 0.005.

**FIGURE 3:**
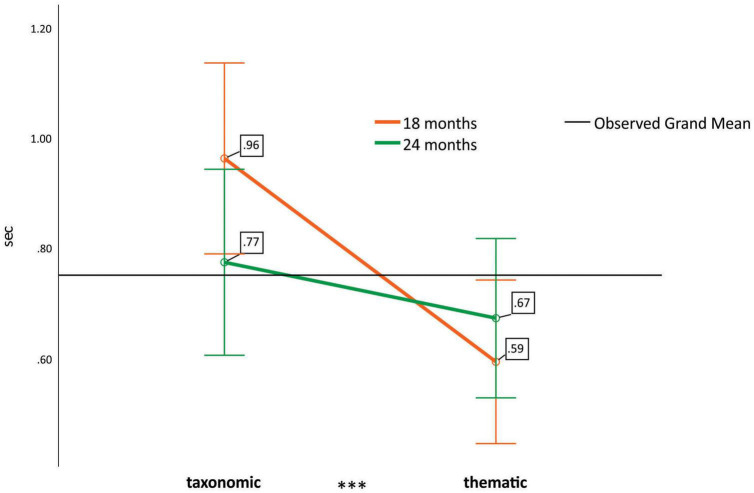
Position-counterbalanced total fixation duration for the two types of categorical distractor pictures in the two age groups. Green line: 18 months; orange line: 24 months; black line: observer grand mean. Error bars represent ± 2 standard errors of the mean. ***p < 0.001.

To examine possible differences in looking times according to category type (taxonomic vs. thematic), we ran a 2 × 2 × 2 mixed ANOVA on the position-counterbalanced total fixation durations on the CD pictures, where the repeated measures variable was category type (taxonomic vs. thematic) and the independent variables were age (18 vs. 24 months) and gestational status (preterm vs. full-term). The significant main effect of category type [*F*(1,43) = 13.051, *p* < 0.001, μ^2^ = 0.233] was caused by the taxonomic relations attracting longer fixations than the thematic relations. Regarding the between-subject variables, there was a significant interaction of category type and age [*F*(1,43) = 4.222, *p* = 0.046, μ^2^ = 0.089], caused by taxonomic preference being present in the younger age group only (18 months) and the opposite pattern being present in the older age group (24 months) (see Figure 3).

**FIGURE 4:**
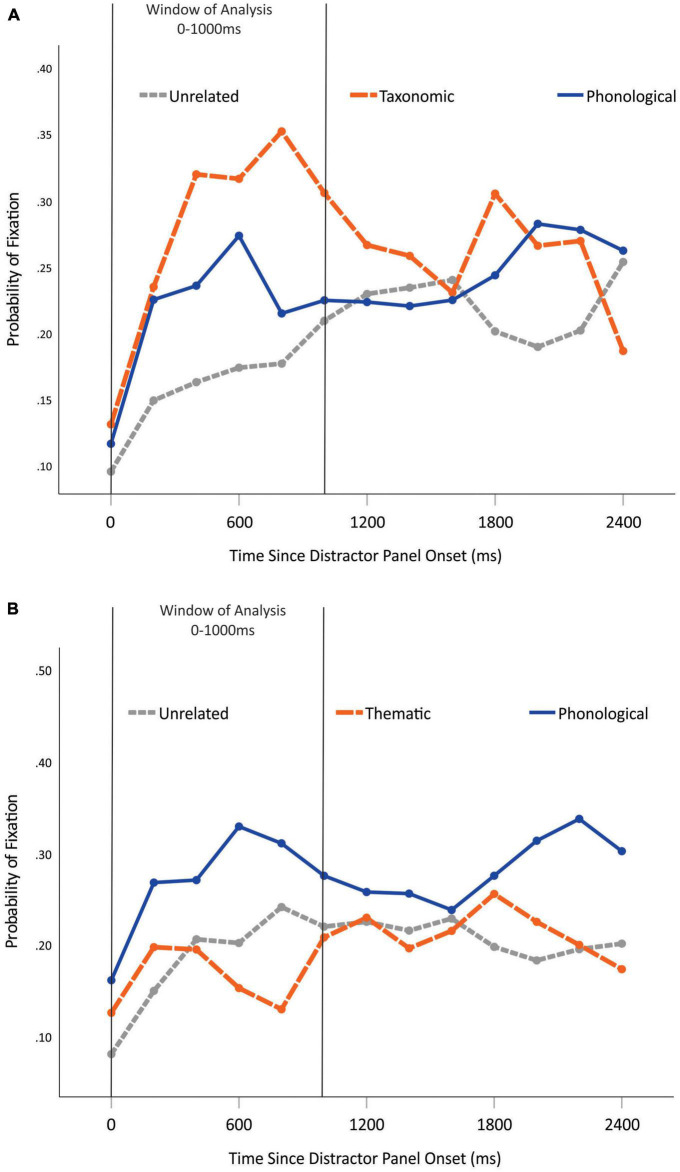
**(A)** Fixation proportions for the unrelated and the taxonomic and phonological distractor picture types during the total fixation period. **(B)** Fixation proportions for the unrelated and the thematic and phonological distractor picture types during the total fixation period. The 0–1,000 ms time window for the statistical analyses is highlighted. Gray dotted line: unrelated picture type; orange dashed line: thematic distractor picture type; blue solid line: phonological distractor picture type.

Fixation probabilities for the first half of exposure post label onset were compared for the three picture types (U, CD, PD) in the two age and gestational status groups. For this analysis, we also used the position-counterbalanced values, to which we applied logistic transformation. We conducted two 6 × 3 × 2 × 2 mixed ANOVAs for the six time bins (0–1,000 ms) with the three picture types (U, CD, PD) as within-subject variables, and with age and gestational status as grouping variables separately for the taxonomic and thematic relations. Figure 4 presents the fixation probabilities separately for the taxonomic (4a) and thematic (4b) relations compared to the U and PD picture types for the whole of the registered time course.

In the case of taxonomic relations, for the main effect of time the Greenhouse-Geisser estimate of departure from sphericity was ε = 0.76. This main effect was significant [*F*(3.79,162.97) = 41.517, *p* < 0.001, μ^2^ = 0.49]. Contrasts revealed that both the 200 and 400 time bin proportions were significantly higher than those of the preceding time bins [0–200: *F*(1,43) = 53.76, *p* < 0.001; 200–400: *F*(1,43) = 18.86, *p* < 0.001]. The main effect of picture type was significant [*F*(2,86) = 12.21, *p* < 0.001, μ^2^ = 0.221]. Contrasts revealed that the U picture type proportions were significantly lower than those of the CD and PD picture types [*F*(1,43) = 24.8, *p* < 0.001].

**FIGURE 5:**
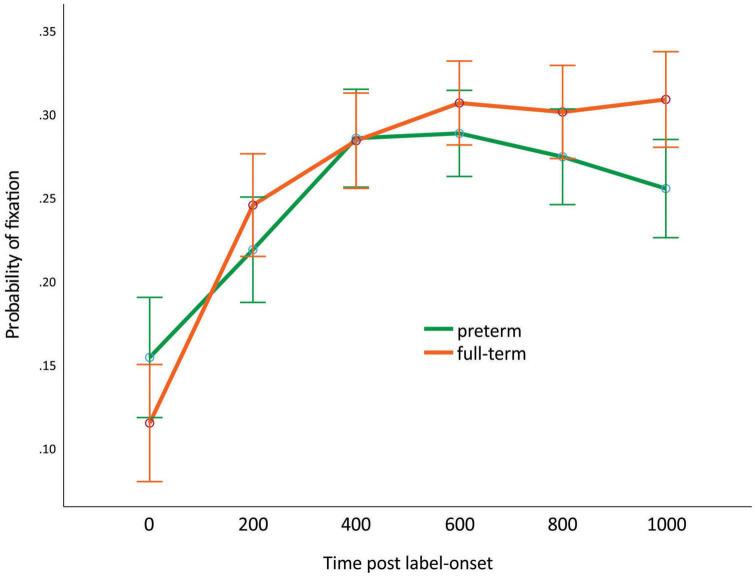
Fixation probability pattern differences between the clinical group and control group in time in the case of the taxonomic relation panels. Green line: preterm; orange line: full-term. Error bars represent ± 2 standard errors of the mean.

With respect to between-subject effects, the interaction of time and gestational status was significant [*F*(3.79,162.97) = 2.65, *p* = 0.038, μ^2^ = 0.058]. Contrasts revealed a significant interaction at 0–200 ms, where only the full-term group’s fixation probability differed in time, while that of the preterm group did not [*F*(1,43) = 6.16, *p* = 0.017, μ^2^ = 0.125; see Figure 5]. However, this contrast yielded a medium effect size. The interaction of picture type and gestational status was significant [*F*(2,86) = 3.17, *p* = 0.047, μ^2^ = 0.069]. Contrasts revealed a significant interaction between the taxonomic and phonological distractors, as the full-term group fixated more on the CD pictures than the PD pictures, while the preterm group fixated on them equally [*F*(1,43) = 4.88, *p* = 0.033, μ^2^ = 0.102].

In the case of the thematic relation panels, for the main effect of time the Greenhouse-Geisser estimate of departure from sphericity was ε = 0.67. This main effect was significant [*F*(3.36,144.56) = 15.84, *p* < 0.001, μ^2^ = 0.27]. Contrasts revealed a significant interaction between 0 and 200 ms, where the fixation probability increased from the baseline level to 24.5% probability on average [*F*(1,43) = 63.52, *p* < 0.001, μ^2^ = 0.6]. For the main effect of picture type, the Greenhouse-Geisser estimate of departure from sphericity was ε = 0.73. This main effect was significant [*F*(1.92,60.05) = 25.84, *p* < 0.001, μ^2^ = 0.37]. Contrasts revealed that the PD picture type proportions were significantly higher than those of the other two types [*F*(1,43) = 21.81, *p* < 0.001, μ^2^ = 0.34]. We did not find any significant interaction or any main effect of the between-subject variables.

## 3. Discussion

The present study tested the lexical-semantic knowledge organization of 18- and 24-month-old participants using the infant-adapted target-absent visual world paradigm for eye tracker developed by Chow et al. (2017). We aimed to identify whether qualitative and quantitative expressive vocabulary or audiovisual capacity differences affect the organization of the mental lexicon when the vocabulary is growing at a rapid rate. If maturational differences affect the organization of the early lexicon, differences should be observed between age-corrected late preterm and full-term control infants in terms of their sensitivity to semantic or phonological relations. To compare early expressive vocabulary capacities we used the Hungarian version of the MacArthur-Bates CDI (Kas et al., 2017). Based on earlier studies that revealed 18-month-old toddlers’ capacity for implicit picture naming or the processing of semantic relations (Mani and Plunkett, 2010; Delle Luche et al., 2014), we hypothesized that a similar organizational pattern would be found to that of the 24-month-old group. To eliminate the methodological inconsistencies of earlier paradigms (cf. Wojcik, 2018), we used a paradigm successfully adapted to infants by means of a modification involving the use of onset syllable–matched phonological distractors and taxonomic and associative semantic distractors distinguished according to animate and inanimate kinds. We expected to find sensitivity to associative semantic relations in the 24-month-olds, in keeping with earlier studies (Rämä et al., 2013; Chow et al., 2017).

Confirming our first hypothesis, our results demonstrated that the 18-month-old infants organized their mental lexicon similarly to the 24-month-olds. We identified phonological and semantic relational effects in both age groups and found that the total fixation durations for the categorical and phonological distractors differed from the unrelated picture types without a significant main effect of age. Previous studies involving toddlers younger than 20 months of age used several forms of the IPL paradigm or the head-turn preference paradigm (Arias-Trejo and Plunkett, 2009; Styles and Plunkett, 2009; Rämä et al., 2013; Borovsky and Peters, 2019). We suppose that the use of the infant-adapted visual world paradigm with a phonological match based on the onset syllable in a language with a highly predictable word-initial stress pattern (Varga, 2002) possibly facilitated the activation of the phonological link between the primes and the phonological distractors in the 18-month-olds. As the acquisition of words (especially nouns and adjectives) utilizes generalization at the superordinate level from the beginning of language acquisition (Waxman and Markow, 1995), we emphasized the importance of the systematic separation of the taxonomic and associative semantic relations by main ontological domain (animate vs. inanimate kinds). We argue that these changes helped us to avoid the earlier methodological inconsistencies summarized by Wojcik (2018). Our results reveal that lexical organization and semantic organization exist in parallel, verifying the findings of earlier network growth models (Hills et al., 2009a).

In accordance with our second hypothesis, the nature of the semantic organization differed between the two age groups: the 18-month-olds were sensitive to taxonomic relations, while the older group looked longer for the thematic relations. This latter effect was verified earlier in a similar visual world paradigm study (Chow et al., 2017). In the younger groups, taxonomic sensitivity to the animate kind with respect to basic-level object naming was predictable based on the conceptual development literature (Tversky, 1989; Waxman and Hall, 1993; Murphy, 2018). The time course analysis verified the strong effect of taxonomic relations on fixations: the proportion of fixations on categorical relations was higher from the beginning. This pattern was missing in the case of thematic relations (manipulable objects), where the phonological distractor received more fixations in general. As motor experience influences attention and categorization behavior (Oakes et al., 1996), the thematic relations may have been strengthened by experience and the increase in self-initiated actions.

Several studies have pointed out that preterm infants, regardless of the severity of prematurity, are at risk of language disorders (Barre et al., 2011; Pritchard et al., 2014). Other studies have emphasized the importance of testing lexical-sematic organization in groups at risk of delayed language development (Borovsky and Peters, 2019). Our analysis of expressive vocabulary size using the CDI revealed that the age-corrected preterm and full-term toddlers acquired lexical categories in a similar order and at a similar rate. The nouns category was acquired first, followed by predicates, while social terms and grammatical words were acquired last in both groups. In line with the study by Stolt et al. (2007) involving Finnish preterm 2-year-olds, our findings also support a universal sequence (Caselli et al., 1995) in the development of lexical categories, independent of gestational status.

Expressive vocabulary size did not predict fixation duration. As the findings of previous studies have not been consistent in terms of the effect of vocabulary size, our results are not without precedent. In a similar paradigm, Chow et al. (2017) found an effect only of receptive vocabulary size on fixation duration. Similarly to our findings, Borovsky and Peters (2019) found a semantic facilitation effect in 18-month-olds with small productive vocabulary.

According to our third hypothesis, we expected that preterm toddlers’ sensitivity to phonological and semantic relations would not be as mature as that of full-term toddlers at the same maturational age. With respect to total fixation duration results, we found no differences between the age-corrected preterm and full-term infants. Our time course analysis revealed a significant interaction between time and gestational status caused by the higher proportion of fixations in the case of the full-term group at between 0 and 200 ms. We detected a slight delay in the preterm group, which reached the maximum proportion of fixations at 400 ms. The significant interaction between picture type and gestational status at the beginning of processing (in the first half of exposure post label onset) also revealed a delay in attention to taxonomic relations. As the total fixation durations did not differ, we can interpret these results as a qualitative strategic difference in processing. As we used gestational correction for the age of the preterm group in our study, this difference in processing reflects a maturational difference.

The strategic difference in processing is in parallel with our result for the interaction between age and preterm status in the acquired number of social terms. Here, the performance of the FT18 toddlers significantly exceeded that of the PT18 toddlers, although this difference was not present at 24 months. The lower number of social words might indicate a more profound problem with social situations in these early months. In their study, De Schuymer et al. (2012) found that late preterm infants at the age of six months showed greater gaze aversion in social and non-social situations. As the shifting of attention is more critical in social situations, this may affect the rate and speed of the acquisition of social words. We emphasize this result as an argument that intact vocabulary growth in this population does not indicate that late preterm infants organize their words in the same manner as their full-term peers. A recent language perception study (Varga et al., 2021) revealed that, despite age correction, late preterm infants process suprasegmental cues in a qualitatively different way from their full-term peers.

Contrary to our expectations, we found a strategic but not a quantitative difference between the fixation patterns of the two groups. We therefore conclude that the lexicon of healthy, age-corrected late preterm infants is organized in the same way as that of full-term controls of a corresponding maturational age. Our results reveal that the development of the semantic network structure is independent of maturational status. Our results also imply that priming is not a difficult audiovisual integration task for healthy preterm toddlers (born without structural brain abnormalities). As the preterm toddlers’ ages were corrected, this correction may have contributed to the equal performance between preterm and full-term toddlers in the priming task and in terms of the results of the CDI.

## 4. Limitations

Our study has several limitations that should be taken into consideration. First, we examined a limited number of full-term and preterm toddlers, as we prioritized the formation of homogeneous experimental and control groups. We recruited late preterm toddlers without any recognizable organic deficits. Another potential limitation is the possible bias of the parents (especially the parents of preterm infants) when completing the CDI test. As the CDI is highly reliable and has good predictive validity (Reese and Read, 2000), and as we obtained similar results to earlier studies, we consider this to be a minor issue. We did not include gender as a moderating variable, due to the small sample size.

## 5. Conclusion and further questions

By addressing certain methodological challenges, our study revealed the stable organization of the lexical-semantic network from 18 months of age. Dissociating taxonomic and associative semantic relations along the main ontological boundary helped the younger age group to successfully detect semantic relations in a similar way to their already demonstrated lexical processing. The 18-month-olds paid greater attention to taxonomic relations in terms of objects from the animate domain, while the 24-month-olds paid greater attention to thematic relations in the domain of manipulable objects. This dissociation reflects an increasing experience with objects and the development of motor skills. Despite age correction in the healthy late preterm group, a strategic difference was detected in the processing of semantic relations. According to our findings, these children still risk facing difficulties at a later stage in terms of their academic achievement.

A question that remains to be explored in future studies is what happens when the number of newly acquired words radically increases. As the lexicon expands, inhibition or attention shifting are more likely to be needed. There is a possibility that at later stages (36 months) we might encounter differences between the preterm and full-term groups due to executive control problems in the former.

## Data availability statement

The datasets presented in this study can be found in online repositories. The names of the repository/repositories and accession number(s) can be found below: https://doi.org/10.18710/HNTTSN.

## Ethics statement

The studies involving humans were approved by the Research Ethics Committee at the Faculty of Education and Psychology of Eötvös Loránd University, Budapest Hungary. The studies were conducted in accordance with the local legislation and institutional requirements. Written informed consent for participation in this study was provided by the participants’ legal guardians/next of kin.

## Author contributions

AR: conceptualization, investigation, formal analysis, methodology, writing—original draft, and writing—review and editing. ZV: conceptualization, investigation, methodology, writing—original draft, and writing—review and editing. MS: funding acquisition, resources, supervision, and writing—review and editing. All authors contributed to the article and approved the submitted version.
