# Supplementary material for: Stable organization of the early lexical-semantic network in 18- and 24-month-old preterm and full-term infants: an eye-tracker study
Source: Front Psychol. 2023 Sep 21;14:1194770. doi: 10.3389/fpsyg.2023.1194770 (PMC10552860; doi:10.3389/fpsyg.2023.1194770)
Supplement: Supplementary file 1 [file Data_Sheet_1.docx]

Supplementary Material

Stable Organization of the Early Lexical-semantic Network in 18- and 24-month-old Preterm and Full-term Infants: An Eye-tracker Study

Anett Ragó*, Zsuzsanna Varga, Miklos Szabo

*** Correspondence:** Anett Ragó: [anett.rago@uit.no](mailto:anett.rago@uit.no)

# Supplementary Figures and Tables

**Supplementary Table 1.** Clinical characteristics of the participants

|  | PT toddlers (n=23) | | FT toddlers (n=24) | |
| --- | --- | --- | --- | --- |
|  | **Median** | **Range** | **Median** | **Range** |
| Gestational Age (weeks) | 35 | 34-36 | 38.5 | 37-41 |
| Birth weight (g) | 2355 | 1770-4000 | 3415 | 2510-4530 |
| Postnatal Age |  |  |  |  |
| 18 months | 19.3 | 19-21 | 18.2 | 17.3-19.1 |
| 24 months | 25.3 | 25.2-26.1 | 24.2 | 23.2-27 |
| Corrected Age |  |  | - |  |
| 18 months | 18.2 | 17.2-19.2 | - |  |
| 24 months | 24.2 | 24-25.1 | - |  |
| Apgar score 1 min | 9 | 0-10 | - |  |
| Apgar score 5 min | 10 | 0-10 | - |  |
| Length of hospital stay (days) | 8 | 4-17 | - |  |
| Days of any Respiration | 2 | 0-6 |  |  |
| TWIN | 13 | 56.5% | 4 | 16.7% |
| Cesarean Section | 17 | 73.9% | 6 | 25% |
| *Neonatal medical complications* | | | | |
| RDS* | 7 | 30.4% | - |  |
| WET LUNG | 1 | 2.1% | - |  |
| Hypoglycemia | 3 | 13% | - |  |
| *Apnea* | 1 | 2.1% | - |  |
| *neonatal Medical Treatment* |  |  |  |  |
| VENTILATION  INVAsive  non- INVASIVE  NONE | 2  12  9 | 8.7%  52.2%  39.1% |  |  |
| Intubated | 3 | 13% |  |  |
| Catecholamines | 2 | 8.7% |  |  |

**Supplementary Table 2.** Stimulus list per panel

| **Panel** | | **Target word** | **Distractor Images** | | | |
| --- | --- | --- | --- | --- | --- | --- |
|  |  | **Phonological Distractor** | **Categorical Distractor** | **Unrelated 1** | **Unrelated 2** |  |
| 1 | **ba**nán *(banana)* | **ba**ba *(baby)* | alma *(apple)*  Taxonomic relation | ágy *(bed*) | madár *(bird)* |  |
| 2 | **ci**pő *(shoes)* | **ci**ca *(cat)* | zokni *(sock)*  Thematic relation | lapát *(shovel)* | kenyér *(bread)* |  |
| 3 | **csi**be *(chicken)* | **csi**ga *(snail)* | kacsa (duck)  Taxonomic relation | lufi (*balloon)* | málna *(raspberry)* |  |
| 4 | **csi**pesz  (*clothes peg*) | **csi**ga *(snail)* | zokni *(sock)*  Thematic relation | motoros *(motorist)* | levél *(leaf)* |  |
| 5 | **ha**jó *(ship)* | **ha**risnya *(tights)* | repülő *(plane)*  Taxonomic relation | lepke *(butterfly)* | csillag *(star)* |  |
| 6 | **ka**bát *(coat)* | **ka**csa *(duck)* | gomb (button)  Thematic relation | szék *(chair)* | fa *(tree)* |  |
| 7 | **ka**kas (cock) | **ka**nál *(spoon)* | tehén *(cow)*  Taxonomic relation | csizma *(boots)* | autó *(car)* |  |
| 8 | **ki**lincs *(handle)* | **ki**fli *(crescent)* | kulcs *(key)*  Thematic relation | maci *(bear)* | csizma *(boots)* |  |
| 9 | **na**drág *(trousers)* | **na**rancs *(orange)* | zokni *(sock)*  Thematic relation | vonat *(train)* | zsiráf *(giraffe)* |  |
| 10 | **sző**nyeg *(carpet)* | **sző**lő *(grape)* | kanapé *(couch)*  Thematic relation | madár *(bird)* | könyv *(book)* |  |
| 11 | **bá**csi *(uncle)* | **bá**rány *(lamb)* | néni *(aunt)*  Taxonomic relation | virág *(flower)* | lufi *(balloon)* |  |

**Note** The English terms are written in italics next to the Hungarian names. The starting syllables are highlighted with boldface type. Note that in Hungarian, the similarly written syllables are pronounced the same way. In the case of the CD, we describe the type of relationship. We used one-, or two-syllable words with two exceptions (kanapé, harisnya, and repülő). These latter are very frequent, three-syllable words.

**Supplementary Table 3.** CDI test categories and their content

| **Main categories** | **Count** | **Word types** |
| --- | --- | --- |
| Social terms | 24 | sound effects and animal sounds |
|  | 30 | games and routines |
|  | 27 | people |
| Common nouns | 18 | vehicles |
|  | 21 | toys |
|  | 81 | food and drink |
|  | 34 | clothing |
|  | 45 | animals |
|  | 36 | inside things |
|  | 53 | outside things and places to go |
|  | 53 | household items |
| Predicates | 138 | action words |
|  | 73 | descriptive words |
| Grammatical function words | 19 | words about time |
|  | 22 | pronoun |
|  | 22 | question words |
|  | 16 | locative |
|  | 16 | quantifiers and articles |
|  | 10 | auxiliary verbs and other word-classes |
|  | 9 | connectives |
|  | 16 | prefixes |

**Supplementary Table 4.** Results of the two-way ANOVAs for the CDI test per age and gestational status

| CDI categories | Main effect | F, p values and µ^2^ |
| --- | --- | --- |
| Vocabulary total score | Age | *F*= 20 (1, 43) *p* < 0.001 µ^2^ = 0.317 |
| Social terms | Age  Age x Status interaction | *F*= 45.97 (1, 43) *p* < 0.001 µ^2^ = 0.517  *F*= 6.32 (1, 43) *p* = 0.016 µ^2^ = 0.128 |
| Nouns | Age | *F*= 18.33 (1, 43) *p* < 0.001 µ^2^ = 0.299 |
| Predicates | Age | *F*= 18.28 (1, 43) *p* < 0.001 µ^2^ = 0.298 |
| Grammatical words | Age | *F*= 15.5 (1, 43) *p* < 0.001 µ^2^ = 0.265 |

**Note** Two-way ANOVAs were performed with the between-subject factors of status (preterm vs. full-term) and age (18 vs. 24 months) to compare the scores and categories of the Hungarian version of the MacArthur Bates Communicative Developmental Inventory.
